# The lifeworld of families of mental health care users in rural South Africa: A phenomenological study

**DOI:** 10.4102/sajpsychiatry.v30i0.2280

**Published:** 2024-11-29

**Authors:** Takalani E. Mbedzi, Anna E. van der Wath, Miriam M. Moagi

**Affiliations:** 1 Department of Nursing, Faculty of Health Sciences, University of Pretoria, Tshwane, South Africa; 2 Department of Nursing, Faculty of Health Sciences, University of Pretoria, Pretoria, South Africa; 3 Department of Nursing, Faculty of Health Sciences, University of Limpopo, Polokwane, South Africa

**Keywords:** caregiver burden, community health care, family member, mental health care users, phenomenology

## Abstract

**Background:**

In recovery-oriented mental health care, family members of mental health care users form part of the caring team. Families are expected to care for mental health care users without support in the under-resourced rural Vhembe district in South Africa.

**Aim:**

This study aims to describe the lifeworld of family members caring for mental health care users in rural areas to inform the development of a support programme.

**Setting:**

Purposive sampling was used to select 16 family members from eight community health centres in the Vhembe district.

**Methods:**

A qualitative approach, using a descriptive phenomenological design, was adopted to conduct unstructured interviews that were transcribed, translated and analysed using a descriptive method.

**Results:**

Family members continuously contemplate their responsibilities. Endless concerns and stress result in forgetfulness and physical problems such as insomnia, hypertension and pain. Family members feel powerless and helpless when there is no improvement and support from community resources. Fear of being violated, embarrassed and stigmatised by community members results in social isolation and depression.

**Conclusion:**

Caregiving is burdensome in poorly resourced areas. Feelings of helplessness and hopelessness Psychosomatic and depressive symptoms relate to the lack of effective community support which are likely to result in compromising the care they provide.

**Contribution:**

The results call for the Department of Health to strengthen community mental health services and for health care professionals to provide supportive interventions based on the needs of mental health care users and their families.

## Introduction

The *South African Mental Health Care Act* defines a mental health care user (MHCU) as a person diagnosed with a mental illness.^1^ Family members of MHCUs are integral members of the caring team in recovery-oriented mental health care. Recovery-oriented care preserves the dignity of MHCUs and families through therapeutic interventions that meet their specific needs.^2^ Family caregivers should be supported by efficient community mental health services to expedite the deinstitutionalisation of MHCUs as stipulated in South African mental health policies.^3^ Deinstitutionalisation relies on the social reintegration of MHCUs.^3^ However, the stigma surrounding mental illness and inadequate mental health literacy hinder successful reintegration and exacerbate the caregiver burden experienced by MHCUs’ families.^3^

South Africa faces a high prevalence of mental health problems. In a countrywide study, 25.7% of respondents reported moderate to severe symptoms of potential depression.^4^ Of the respondents who experienced adverse childhood experiences (associated with a higher possibility of depression and anxiety), 24.9% resided in rural areas.^4^

Primary health care providers should support and encourage family members to oversee MHCUs and monitor their treatment adherence.^5^ Unfortunately, there is a lack of qualified mental health practitioners, effective referral protocols, appropriate infrastructure and reliable access to psychotropic medicines in community-based mental health services in South Africa.^6^ These inadequacies are more evident in rural areas where access to mental health care and treatment alternatives are restricted by various factors such as the concentration of mental health professionals in urban areas.^7^

A systematic review indicated that almost one-third of caregivers experienced a burden of care.^8^ Caregivers with an increased care burden experience more mental health problems and often neglect their own physical and psychological needs.^8,9^ Studies in rural areas in South Africa indicated the unique challenges experienced by caregivers of MHCUs. In Kwa-Zulu-Natal, the burden of care included psychological and socio-economic aspects and inadequate access to supportive resources.^10^ The study demonstrated a gap in aligning service delivery plans to the needs of family caregivers.^10^ Caregivers in the North West reported a high degree of stigmatisation, and the study indicated a gap in collaborative health care efforts to reduce stigma and caregiver burden.^3^

Mental health providers should understand caregivers’ burden of care to advocate for the inclusion of appropriate psychosocial support, especially when families have limited access to services and are living in poverty.^11,12^

Vhembe district in the northern part of Limpopo province is one of the most under-resourced rural districts in South Africa with 67% of adults living in poverty.^13^ Families are expected to care for MHCUs after hospitalisation without support and resources. The first author (hereafter referred to as ‘the researcher’), a mental health care practitioner, identified a need to describe the lifeworld of family members who are taking care of MHCUs in a resource-poor rural area to inform the development of a support programme. The programme has the potential to improve mental health services and support for families in rural areas of South Africa.

## Research methods and design

A qualitative, descriptive phenomenological design^14^ was adopted to answer the research question: What are the lived experiences of family members caring for MHCUs in the Vhembe district? The representation of the family members’ lifeworld experiences is a result of the researcher’s and participants’ dialogical interactions.^14^

### Study setting

Vhembe district consists of four municipalities, namely, Thulamela, Makhado, Musina and Collins Chabane.^13^ There are 112 clinics, 8 community health centres, 6 district hospitals, 1 regional hospital and 1 specialised psychiatric hospital in the district. Eight community health centres were selected for the study because a high number of MHCUs make use of their services (see Table 1).

**TABLE 1:** Average monthly mental health care users’ visits to community health centres in Vhembe district.

Community health centre	Average MHCUs
Thohoyandou	104
Bungeni	127
Tiyani	65
Mphambo	30
Makhado	72
Mutale	75
William Eddie	80
Tshilwavhusiku	95

MHCU, mental health care user.

There is only one psychiatrist in the district who visits the hospitals every month.^15^ The infrastructure of most health facilities in Limpopo is dysfunctional and inappropriate for the special needs of MHCUs.^16^

### Study population and sampling

The research population was selected purposively as they possessed the common characteristics of interest to the researcher.^17^ The accessible population was all family members caring for MHCUs attending monthly follow-up visits at the community health centres. The researcher arranged a date with the clinic manager and recruited study participants who accompanied their family members for follow-up visits. The researcher also requested the nurses to refer potential participants to contact her telephonically.

The inclusion criteria included all family members aged 18 years and older, living and caring for a relative diagnosed with mental illness according to the DSM 5^18^ for a minimum period of 2 years, able to express themselves either in English, Tshivenda, Tsonga or Sepedi and who gave voluntary informed consent.

### Data collection

The researcher collected the data (a female lecturer with a master’s degree) through unstructured face-to-face individual interviews^17^ with family members caring for MHCUs. One pilot interview was conducted, and no changes in the interviewing techniques were made. Before the interview, rapport was established with participants, who were not known by the researcher. The interview guide included the broad interview question: ‘What are your experiences in caring for an MHCU?’ Further probing questions were based on the participants’ responses. The interviews were conducted on a date and time convenient to participants at their homes in a private place with only the interviewer and participant present. Interviews were conducted from April to June 2021, lasted 30–45 min and were audio recorded. Field notes were taken during and after the interview.^17^

After data saturation was reached at participant number 14, two additional interviews were conducted to confirm data saturation.^17^ One participant dropped out because of personal reasons. The interviews were transcribed verbatim and translated into English by a language practitioner proficient in Tshivenda, Xitsonga and Sepedi.

### Data analysis

To capture and explicate the essence and the meaning units constituting the experiences of family members, the following manual coding steps were followed: (1) read all interviews to develop a sense of the whole, (2) develop meaning units for each participant’s experience, (3) cluster relevant units of meanings, (4) develop narrative descriptions for each participant, (5) search for essential structures that could express the entire narrative description, (6) evaluate the description and (7) synthesise the structure from all participants’ accounts.^14^

### Trustworthiness

Credibility was ensured through prolonged engagement with participants until data saturation was reached.^17^ Transferability was enhanced through a detailed description of the research setting.^17^ An independent coder was used to analyse the data, and consensus was reached by the three authors on the results.^17^ As the researchers are mental health care nurse specialists, they bracketed their perceptions of the caregiving experiences of families of MHCUs to ensure confirmability and dependability.^17^ The transcripts and findings were not returned to participants for verification because of logistical reasons.

### Ethical considerations

Ethical clearance was granted by the University of Pretoria Research Ethics Committee (No. 674/2020). Permission to conduct the study was obtained from the Department of Health, Limpopo province (No. LP_2020_12_005) and Vhembe district (S5/6). Ethical principles, such as the right to privacy, confidentiality, autonomy and voluntary participation, were adhered to.^17^ The participants signed informed consent forms explaining the study purpose and procedures, that interviews would be recorded and that they could withdraw from the study at any time. Code numbers were used to identify participants, e.g., *P1* to maintain anonymity. Password-protected transcripts will be saved for 15 years.

The indirect benefits of participating in the study included recommendations to improve support for family members. Although a psychologist was available to counsel participants experiencing potential distress during the interviews, no immediate interventions were needed. The researcher secured counselling appointments for participants who required emotional support.

## Results

The sociodemographic profile of participants is presented in Table 2. The majority of the participants were females, with five participants being male. Their ages ranged between 20 and 74 years. Most participants were unemployed or pensioners with only one permanently employed and one self-employed. The years of care provision varied between 4 and more than 20 years.

**TABLE 2:** Participants’ sociodemographic profile.

Participant number	Gender	Age	Marital status	Occupation	Relationship to the MHCU	Years of care provision	Level of education
1	F	42	Single	Nurse	Daughter	21	Tertiary
2	F	40	Married	Self-employed	Wife	15	Secondary
3	F	59	Married	Unemployed	Sister	10 >	Non-educated
4	F	76	Widow	Old age pensioner	Mother	10 >	Non-educated
5	M	28	Single	Unemployed	Brother	20	Tertiary
6	F	69	Married	Old age pensioner	Mother	10	Primary
7	F	26	Single	Internship	Sister	4	Tertiary
8	M	63	Widower	Retiree	Father	10 >	Tertiary
9	F	63	Single	Retiree	Sister	20>	Secondary
10	M	20	Unmarried	Scholar	Grandson	10 >	Secondary
11	M	74	Married	Old age pensioner	Father	10 >	Primary
12	F	72	Married	Old age pensioner	Mother	10 >	Non-educated
13	F	79	Widow	Old age pensioner	Mother	10 >	Non-educated
14	F	65	Widow	Retiree	Mother	19 >	Tertiary
15	M	57	Widow	Unemployed	Mother	20 >	Primary
16	F	67	Widow	Old age pensioner	Mother	20 >	Non-educated

MHCU, mental health care user; F, female; M, male.

The results are first presented through the essence of the experience, followed by the meaning units that make up the essence (see Table 3). Verbatim participants’ quotes are presented with the participant number and relation to the MHCU in brackets.

**TABLE 3:** Findings.

Essence	Meaning units
The burden of caring for a mental health care user	Consistently contemplating caregiving responsibilities
	Stress-related physical problems
	Feeling helpless and powerless
	Fear of being embarrassed by the mental health care user
	Fear for safety
	Feelings of depression and sadness

### The essence of the phenomenon: The burden of caring for a mental health care user

Family members caring for MHCUs were continuously thinking about their responsibilities. Endless concerns and stress resulted in forgetfulness and physical problems such as insomnia, hypertension and pain. Family members felt powerless and helpless when they observed no improvement in MHCUs’ conditions with limited support from community resources. They felt embarrassed and feared social stigmatisation, resulting in social isolation. Family members feared for their own and the safety of the MHCUs. The lonely struggles gave way to depression and sadness. The essence is further described through the following meaning units as indicated in Table 3.

### Consistently contemplating caregiving responsibilities

Family members were so preoccupied with the caregiving responsibilities that they experienced disturbed thought patterns. A participant shared her fears that her sister might get lost as she sometimes leaves the home unnoticed. She called her sister’s daughter persistently to find out if the MHCU is safe:

‘… when she has relapsed, she cannot sit down, she will be pacing up and down, roaming around, and leaving home. It pains me a lot as I constantly get worried about her … I was always phoning her daughter who is 19 years old because I am afraid that she can leave home without being noticed so that she keeps an eye on her so that she does not escape at night. It will be difficult to trace her whereabouts …’ (P3 sister, F, 59, Married)

The same participant revealed that the emotional distress caused forgetfulness:

‘… I feel that my mind is also not working well during that time. If I can go out in the sun and only to find that I happen to forget something I wanted to take. I fail to understand it …’ (P3 sister, F, 59, Married)

Another participant requested counselling as she could not stop thinking about the possibility of her sister harming herself or someone else:

‘Even counselling I need it because we are thinking too much. Sometimes she can decide to kill herself or kill us … Counselling will help us to refrain from persistent memories of this entire thing ….’ (P7 sister, F, 26, Single)

### Stress-related physical problems

Participants described how the constant burden of care and associated stress resulted in physical problems such as hypertension and emotional distress:

‘… I am taking pills for blood pressure. Okay, there are times when you look and find that it can cause your blood pressure to go up.’ (P14 mother, F, 65, Widow)

Another participant experienced muscle pains and a lack of energy. The health care providers at the clinic diagnosed the condition as stress related:

‘Then I went to the clinic, they said you are suffering from stress related problems, my head was so heavy, neck pains experienced, lack of energy, so they said that its stress. They even asked me what is bothering me.’ (P16 mother, F, 67, Widow)

One participant reported altered sleeping patterns as he was constantly wondering how he would deal with all the caregiving responsibilities:

‘You cannot sleep when someone is sick, you may sleep for five minutes, three hours or two hours. You will be thinking … how I am going to do it? Even if I can tell myself that I am going to take him to the hospital, where will I get the money to pay for transport … who can offer it free of charge? No one will do that. If he becomes violent and when his condition deteriorates, who will assist me to apprehend him, am I going to call the relatives to help me?’ (P5 brother, M, 28, Single)

### Feeling helpless and powerless

Some participants reported feelings of helplessness when they had to manage the challenging behaviours displayed by MHCUs without help. The following quotation depicts the reluctance of police members to assist families:

‘There was a certain time when my mother went there and sat there [*at the police station*] for the whole day and was told that the police van was not available … my sister visited them and was also told the same story of the unavailability of the police van … Only to find that the officers have agreed amongst themselves that this person [*MHCU*], they cannot manage to apprehend him.’ (P4 brother, F, 76, Widow)

Lack of community support contributed to feelings of powerlessness. Participants received no support from the community when they needed to apprehend the MHCU during relapse. An elderly mother felt helpless when the community members failed to assist during a crisis:

‘That’s bringing pressure on you since the patient is not well, he has relapsed from his mental condition, when you look at the community members they just watch or ask: “What is happening?” … [*that*] brings a lot of frustration to us as a family.’ (P4 brother, F, 76, Widow)

‘Because people around this village they are afraid of her, even if I scream, “Yo, Yo help me!” they do not respond, she can rather kill me … because she is known to beat people … it becomes a problem as I am alone fighting with her.’ (P16 mother, F, 67, Widow)

Family members felt helpless and sad when they observed no improvement in the MHCU’s condition:

‘I am not someone who is free, who is healthy, I can only be healthy when I see my patient being healthy … I can only be happy if my patient is looking happy. But if she is not well … me too, I also get ill and feel so helpless.’ (P12 mother, F, 72, Married)

Participants accepted responsibility for the caregiving role, but experienced helplessness when no one was willing to relieve them of the caregiving burden, for example, in the event of work responsibilities:

‘… we are all affected, and this causes depression … what are we going to do? Nothing really, during the relapse episode, if you are working somewhere far from home, with whom are you going to leave him, and who will provide care?’ (P5 brother, M0, 28, Single)

### Fear of being embarrassed by the mental health care user

The stigma and negative remarks from community members caused embarrassment as in the following two statements:

‘When you go out there in the community it is painful and embarrassing because people usually pass the remarks that you are not taking care of the patient.’ (P9 sister, F, 63, Single)

‘That brings pressure on you since when the patient is not well, he has relapsed from his mental condition, when you look at the community members they just watch or ask what is happening. All that I have indicated brings a lot of frustration and embarrassment to us as a family.’ (P5 brother, M, 28, Single)

One participant feared that she would be embarrassed when the MHCU presented with confusion and inappropriate behaviour after epileptic seizures:

‘After she had epileptic fits, she would run away, and undress clothes in public … When she is doing all that, other kids will be watching … and that worries me a lot in my life, that is my worst pain.’ (P3 mother, F, 59, Married)

A participant feared leaving the MHCU alone, but, on the other hand, anticipated that the MHCU might embarrass her in public, for example, when attending a funeral:

‘She cannot go [*to the clinic*] alone, I need to accompany her since we have been told that we should always accompany them … I sleep in the same room with her. When I attended the funerals … I used to go with her. I am usually afraid that she may behave in an unacceptable manner in the public eye at the funerals … I then decided to leave her but I needed to come back home early immediately after the funeral.’ (P4 mother, F, 76, Widow)

### Fear for safety

Most of the participants feared the verbal and physical aggressions displayed by the MHCUs. Especially female and elderly caregivers felt powerless to manage aggressive behaviour. A participant shared her experiences of living in fear of being attacked by the MHCU:

‘… at times when I am seated alone here, I would start [*to*] think that if she can relapse and find me seated relaxed like this when we are only two in the house. What will happen? Definitely, she will kill me. I am always scared … When you look at me [*removing the mask and showing the scars on her face where she was bitten*]. She had bitten me when I wanted to apprehend her the time, she wanted to beat me up. She is very aggressive, fights a lot, beats people.’ (P16 mother, F, 67, Widow)

Another participant tried to cope with the unprovoked aggression and fear through prayer:

‘… she wants to fight us, she swears at me while she can find me seated not doing anything. I said I will see how far she can go. I prayed telling myself that I am not going to be affected by this situation.’ (P7 sister, F, 26, Single)

An elderly mother expressed fear related to concerns for the MHCU’s safety. She feared that the MHCU would become a victim of sexual abuse or human trafficking:

‘Holding people hostage, human trafficking, people are raped … This is the reason I am saying it is really bad out there in this world … Hear the news that a child disappeared when searched around and could not be found anywhere. I am very scared that the same can happen to my patient.’ (P16 mother, F, 67, Widow)

### Feelings of depression and sadness

Symptoms of depression were triggered by the caregiving burden. Participants experienced feelings of sadness, social isolation, constant concerns and suicidal ideas. One participant explained how the stress caused feelings of despair and emotional exhaustion:

‘Nothing, how can I get help, nothing. This is just a challenge, which I just look at it. My daughter is not staying far from this place she told me one time that this pressure is too much for me to handle, it will make me be affected psychologically and suffer from depression. I told her that deep inside I feel emotionally drained so much.’ (P4 mother, F, 76, Widow)

Another participant shared her feelings of despair and sadness when her sister refused medication and meals because of persecutory delusions:

‘Sometimes when I cook food, she will say I am not going to eat your food because you poured poison in the food … at times I will find myself crying … She did not want to use her medication. She would throw away the pills as she believed that she is not mentally ill and furthermore holding the belief that I wanted to kill her … I cry a lot ….’ (P7 sister, F, 26, Single)

In one participant, the burden of care resulted in suicidal thoughts, while another experienced disappointment caused by the onset of the mental illness, which destroyed the future of the MHCU:

‘Sometimes as am thinking over and over again, I also think of committing suicide, so that I can kill myself.’ (P10 grandson, M, 20, Unmarried)

‘It is painful because he passed matric [*grade 12*] being well, then when you see him being mentally ill … therefore counselling is mandatory. As you expected him to go to the university … it is painful really.’ (P5 brother, M, 28, Single)

## Discussion

The findings provide a glimpse into the lifeworld of family caregivers in a resource-poor area in South Africa. The phenomenological approach illuminated the family members’ spiralling into depression as they were left helpless with persistent fear and concerns. Family caregivers in various African countries also identified themes of exhaustion, hopelessness, helplessness, shame, fear, social challenges and inadequate attention to their own support needs.^19^

The study participants were concerned about the lack of support to relieve them from the caregiving burden and MHCUs’ inadequate improvement. Similarly, caregivers in Nigeria worried endlessly about the prognosis of the MHCU.^11^ In Malaysia, caregivers expressed concerns that no one would take care of the MHCU after their death.^20^ Caregiver concerns and stress are more intense among older female caregivers who are parents to the MHCU and caregivers from lower socioeconomic status.^12,21^

The physical burden of care is reported in various studies. It is exacerbated when family members do not have time to rest, lose energy, experience sleeping disorders and neglect their health.^20,22^

The participants in this study felt helpless to cope with the unacceptable behaviour of MHCUs. Caregivers felt helpless and powerless when the condition of the MHCU worsened despite their best efforts in care provision.^23^ Caregivers experienced shock, sadness, depression, exhaustion and an inability to comprehend the situation. Feelings of helplessness occurred during crises.^24^ In response to the psychological distress, participants wished to be given hope by health care providers.^25^ Participants felt powerless when community and police officers refused to help them in a crisis. In a similar study, fear of MHCUs who were believed to be dangerous hampered community support.^26^

Family caregivers felt embarrassed because society looked down on them and insulted the MHCUs.^19^ In this study, participants attributed feelings of embarrassment to the unpredictable behaviours of MHCUs in public. Resorting to social isolation added to the psychological burden, similar to a study that attributed caregivers’ emotional distress to the stigma towards mental disorders.^20^ Stigma is associated with psychological, social and intrapersonal consequences and is triggered by misconceptions about mental illness, resulting in discrimination.^27,28^ Stigmatisation may result in low self-esteem, feelings of shame, social isolation, high levels of stress and reluctance to seek professional help.^19,29^

Family members experienced fear for their safety and that of the MHCUs. Aggressive behaviour of MHCUs was mentioned as a family concern in many studies.^12,24,30,31^ Family members who were concerned about the MHCU’s safety felt obliged to supervise the MHCU continuously.^20^

Depression in caregivers presented as self-blame, hopelessness and concerns about the future.^23^ Caregivers’ depression may have various causes, such as MHCUs who are suicidal, seeing the MHCU suffer and self-blame.^20,30^ Participants in this study were constantly thinking about their caregiving responsibilities, similar to a study in Zimbabwe, where participants linked the phenomenon of ‘thinking too much’ with feelings of depression.^25^

### Strengths and limitations

Before the actual data collection, the researcher visited the study participants to build rapport and establish mutual understanding and respect, making the participants feel at ease in sharing their lived experiences. The data collection was done through face-to-face interviews at the participants’ homes, allowing the researcher to observe non-verbal cues such as facial expressions.

Only family members of MHCUs who followed up at the clinics were referred by the nurses for potential inclusion in the study. Family members of non-compliant MHCUs might have provided different caregiving experiences. Another limitation was that the transcripts and findings were not verified by the participants. The inclusion criteria did not quantify the duration of care in measurable terms, but all participants were providing care for extensive periods of as long as 20 years.

## Conclusion

The findings confirmed that caring for MHCUs is burdensome, especially in poorly resourced rural villages in South Africa. Participants shared physical, emotional and cognitive burdens associated with caregiving experiences. Some participants, mostly female participants, struggled to deal with feelings of helplessness and hopelessness because of ineffective community support, even from community structures such as police services. Considering the psychosomatic symptoms and feelings of depression, family members are likely to suffer from mental illness, compromising the care they provide. The results call for the Department of Health to strengthen community mental health services so that health care providers can implement psychosocial interventions such as support groups, family therapy and crisis interventions. Interventions should be based on the needs of MHCUs and their families. The authors used the findings to develop a psychoeducational support programme for family members.
